# A case of emergent operation for a life-threatening infectious mediastinal cyst

**DOI:** 10.1016/j.ijscr.2019.10.022

**Published:** 2019-10-18

**Authors:** Daisuke Taniguchi, Tomoshi Tsuchiya, Keitaro Matsumoto, Takuro Miyazaki, Go Hatachi, Koichi Tomoshige, Ryoichiro Doi, Hironosuke Watanabe, Yoshiaki Zaizen, Junya Fukuoka, Takeshi Nagayasu

**Affiliations:** a Department of Surgical Oncology, Nagasaki University Graduate School of Biomedical Sciences, Nagasaki, Japan; b Medical-Engineering Hybrid Professional Development Program, Nagasaki University Graduate School of Biomedical Sciences, Nagasaki, Japan; c Department of Pathology, Nagasaki University Graduate School of Biomedical Sciences, Nagasaki, Japan

**Keywords:** Mediastinal tumor, Emergent operation, Bronchogenic cyst

## Abstract

**Purpose:**

Congenital mediastinal cysts are an uncommon but important diagnostic group. Most of these cysts are benign and asymptomatic in adults. However, some of them are clinically problematic due to the compression of neighboring organs, infection, or perforation.

**Case presentation:**

A 20-year-old man presented with severe dyspnea. Imaging revealed a mediastinal cyst in the subcarinal space compressing his right pulmonary artery and airway, which was later diagnosed as a bronchogenic cyst. Due to quick symptom exacerbation, emergent cyst wall fenestration was performed through video-assisted thoracic surgery with “stand-by” extracorporeal membrane oxygenation. Complete cyst resection was difficult owing to adhesion of the cyst to the surrounding organs. The symptoms immediately resolved after surgery and the postoperative course was uneventful.

**Conclusion:**

Mediastinal bronchogenic cysts with life-threatening complications are rarely reported in adults. However, this case was life-threatening due to airway and vascular compression; emergent surgical care should be considered in such cases.

## Highlights

• Congenital mediastinal cysts are an uncommon but important diagnostic group.
• Mediastinal bronchogenic cysts with life-threatening complications are rarely reported in adults.
• The patient’s condition can be emergent due to airway and vascular compression in some cases.
• We considered that the reason for this acute exacerbation was cyst bleeding because a two-layered cyst was observed on MRI.
• Emergent surgical care should be considered in such life-threatening cases.

## 1 Introduction

Congenital mediastinal cysts are an uncommon but important diagnostic group, representing 12–18% of all mediastinal masses [1]. Mediastinal cysts are regarded as congenital abnormalities that can occur in infants, children, and adults. The classification of mediastinal cysts is based on their etiology, encompassing bronchogenic, esophageal duplication cysts of foregut origin, mesothelial derived pericardial/pleural cysts, thymic cysts, and other miscellaneous cysts. Most of these cysts are benign and asymptomatic in adults. However, some of them are clinically problematic due to the compression of neighboring organs, infection, or perforation [1]. We present a case of emergent operation for a life-threatening mediastinal cyst. This report was prepared according to the SCARE guidelines for reporting surgical case reports [2].

## 2 Case report

**Fig. 1:**
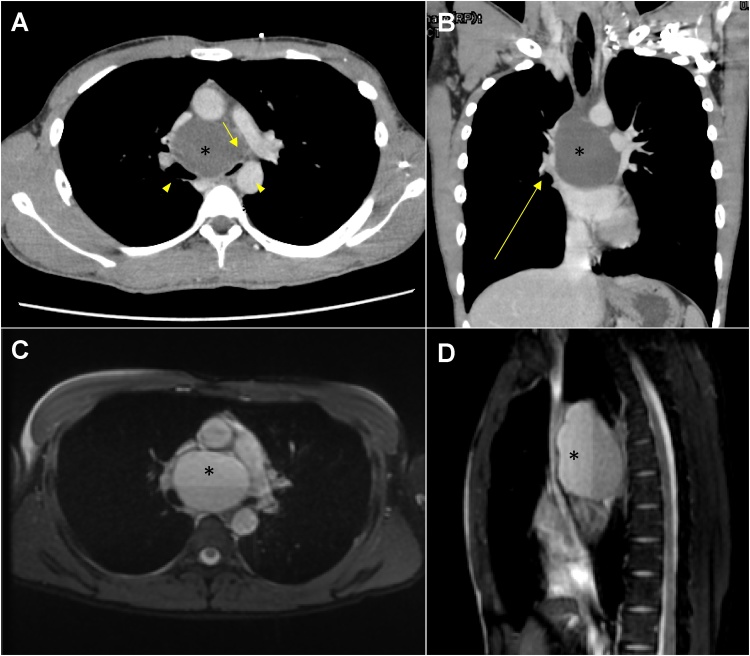
Preoperative images. A, B. Chest computed tomography. A mediastinal mass was noted in the subcarinal space (*). Compression of the right pulmonary artery (short arrow) and airway (arrowhead). Delayed enhancement of the right pulmonary vein and airway was seen at the tracheal bifurcation (long arrow). (A: Axial, B: Coronal). C, D. Fat-suppressed T2-weighted magnetic resonance imaging. A two-layered simple cyst (*) was observed, which indicated infection or bleeding inside the cyst. (A: Axial, B: Sagittal).

**Fig. 2:**
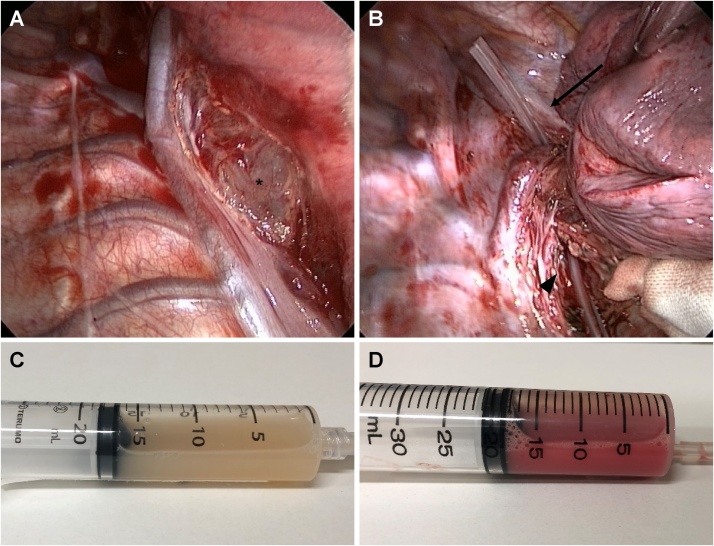
Operative findings. A. The cyst wall appeared on the subcarinal lesion (*) after the dissection of mediastinal pleura. B. We inserted a drainage tube through the cyst after cyst wall fenestration on subcarinal lesion (arrowhead) and superior mediastinum (arrow). C. Aspirated white pus after cyst puncturing. D. Fluid inside the bottom layer of the cyst. Both pus and blood were found.

**Fig. 3:**
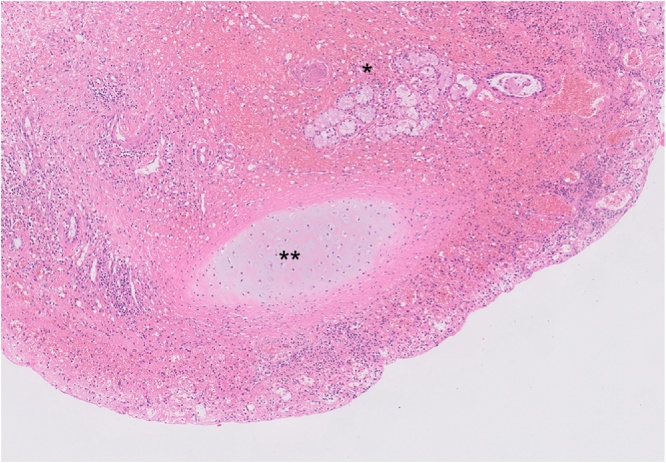
Pathological findings of the cyst wall (hematoxylin and eosin stain). Bronchial gland (*), cartilage (**) with infiltration of inflammatory cells.

A 20-year-old man presented with mild dyspnea and was diagnosed with a common cold at the first visit to a clinic. However, severe dyspnea persisted after 3 days. Enhanced computed tomography (CT) scan revealed a mediastinal mass in the subcarinal space, which compressed the right pulmonary artery and a delayed right pulmonary vein and airway enhancement at the tracheal bifurcation (Fig. 1-A, B). The mass was diagnosed as a mediastinal cyst and bronchoscopy was attempted; however, the patient was unable to be placed in the supine position due to severe respiratory distress. The patient was then transported to our hospital by ambulance due to the need for emergent surgery. Despite the fact that transportation took only two hours, his white blood cell count increased rapidly and symptoms became progressively worse, which suggested that the patient’s condition was emergent. Signed consent was obtained for all procedures. To ensure that the mass was a simple cyst without septum and mural nodule, magnetic resonance imaging (MRI) was performed as the patient’s condition allowed; the image showed a two-layered simple cyst, which indicated an infection or bleeding inside the cyst (Fig. 1-C, D). Since the symptoms developed and exacerbated rapidly, we performed emergent surgery. After the induction of general anesthesia with “stand-by” extracorporeal membrane oxygenation, video-assisted thoracic surgery (VATS) was performed. First, we punctured the cyst and aspirated white pus; then, we performed cyst wall fenestration on the subcarinal lesion and superior mediastinum. The bottom layer fluid inside the cyst contained both pus and blood, which were compatible with MRI findings, while continuous bleeding from the cyst wall was not observed (Fig. 2). Performing complete cyst resection was difficult owing to severe adhesion of the cyst to surrounding organs such as both main bronchi and pericardium. After surgery, the symptoms resolved immediately and completely. The postoperative course was uneventful and the patient was discharged on the 15^th^ postoperative day. Pathological examination of the cyst wall revealed an inflamed bronchogenic cyst with the findings of bronchial gland, cartilage with infiltration of inflammatory cells, and no malignancy (Fig. 3). Six months after the operation, no sign of recurrence was observed.

## 3 Discussion

Bronchogenic cysts are rare malformations that usually develop in the tracheal branches of the mediastinum or the pulmonary parenchyma. Most bronchogenic cysts are diagnosed incidentally because patients usually have no symptoms [3]. The usual presentation of a mediastinal bronchogenic cyst is related to cyst infection and/or tracheobronchial compression. Life-threatening adverse events are rare [3]. In addition, mediastinal bronchogenic cysts with life-threatening complications are rarely reported in adults; most occur in infants and children [4,5]. Subcarinal cysts account for 7–28% of all mediastinal bronchogenic cysts [5]. Their symptoms develop due to extrinsic compression of the tracheal bifurcation, left atrium, and other adjacent organs [4]. The present case showed a subcarinal cyst without any of the above symptoms before the current episode; however, acute symptom progression was observed. We considered that the reason for this acute exacerbation was cyst bleeding because a two-layered cyst was observed on MRI and the bottom layer fluid inside the cyst contained both pus and blood, as observed intraoperatively.

Cyst margin removal is controversial, and complete resection should be sought to avoid recurrences [3]. If the cyst is large and is compressing the pulmonary artery, gradual aspiration of the cystic fluid before extraction of the cyst is mandatory to prevent hemodynamic complications, such as reperfusion lung injury [4,6]. Thoracotomy has been described as superior to minimally invasive procedures to remove pericystic adhesions [3]. Increasing experience with VATS has demonstrated its safety and effectiveness in the resection of bronchogenic cysts [3], although conversion to thoracotomy has been described in 3.5% of VATS procedures [3]. Because of dense pericystic adhesions to adjacent organs, surgical excision of mediastinal bronchogenic cysts can be hazardous [7]. Major operative difficulties or intraoperative complications were reported in 43.9% of cases, all of which were symptomatic preoperatively [7]. Recently, the potential therapeutic role of endoscopic ultrasound-guided fine-needle aspiration in infected mediastinal bronchogenic cysts has been reported [8]. In this case, symptoms developed and exacerbated rapidly so we selected cyst wall fenestration by VATS under stand-by extracorporeal membrane oxygenation because we focused on the life-saving aspect rather than reducing invasiveness or complete resection. The aspiration of the intracystic fluid before fenestration might be effective to prevent hemodynamic complications. Since severe adhesions to neighboring organs were expected and no residual cystic lesion was seen on CT after surgery, we decided to avoid a second-look operation. Careful and long-term follow-up will be needed to detect recurrent swelling since recurrent cases after incomplete surgical resection have been reported [4,6]. If a recurrence occurs, we will plan the cyst resection before the appearance of symptoms.

## 4 Conclusion

Mediastinal bronchogenic cysts can show rapid symptom exacerbation and be life-threatening due to the airway and vascular compression; an emergent surgical approach should be considered in such cases.

## Source of funding

This study was not supported by any grant.

## Ethical approval

Not applicable.

Our ethics committee considers that this is not required for a case report according to the Japanese guidelines for research in humans.

## Consent

The study patient provided signed informed consent for publication of the case details.

## Author contribution

Study concept or design; Daisuke Taniguchi, Tomoshi Tuchiya, Keitaro Matsumoto.

Data collection; Takuro Miyazaki, Go Hatachi, Ryoichiro Doi, Hironosuke Watanabe.

Data analysis or interpretation; Yoshiaki Zaizen, Junya Fukuoka.

Writing the paper; Daisuke Taniguchi, Koichi Tomoshige, Takeshi Nagayasu.

## Registration of research studies

NA.

## Guarantor

Daisuke Taniguchi.

## Provenance and peer review

Not commissioned, externally peer-reviewed.

## Declaration of Competing Interest

There are no conflicts of interest to declare.
